# Theory of mind: a new perspective on the puzzle of belief ascription

**DOI:** 10.3389/fpsyg.2015.01184

**Published:** 2015-08-14

**Authors:** Gabriella Airenti

**Affiliations:** Department of Psychology, Center for Cognitive Science, University of Torino, Torino, Italy

**Keywords:** theory of mind, intersubjectivity, false belief, deceit, irony

## Abstract

The concept of theory of mind (ToM) has considerably changed since its first proposal. The aim of first human studies was to understand how young children acquire the representation of others’ mental states, in particular beliefs, and how they distinguish them from their own and from reality. The False Belief Task was designed to prove the acquisition of this capacity. According to children’s performance in this test the acquisition of ToM has been attested at around 4 years of age. In last years it has been shown that using spontaneous response tasks also 15-month-old-children could attribute to an agent a false belief about the location of an object. These results have generated the puzzle of belief-ascription: Why do 3-year-old children fail the classical false belief tasks whereas much younger children show the correct expectation in the spontaneous response tasks? In this paper I shall argue that (i) infants and young children, when confronted with the two forms of false belief tasks do not face the same problem and (ii) behind the two testing situations there are different ways to understand theory of mind. I shall propose that what appears in infants is the natural human disposition to intersubjectivity.

## Introduction

The concept of Theory of Mind (ToM) has considerably changed since its first proposal in the paper Premack and Woodruff’s (1978). Focusing the interest on humans and in particular on human acquisition has posed methodological problems, which are still at issue.

The aim of first human studies was to understand how young children acquire the *representation* of others’ mental states, in particular beliefs, and how they distinguish them from their own and from reality. To test the acquisition of this main conceptual change Wimmer and Perner (1983) designed the False Belief Task. According to children’s performance on this test the acquisition of ToM has been shown to emerge at around 4 years of age.

In recent years a new trend emerged: researchers have found ways to verify the capacity of passing the false belief test in much younger children. Clements and Perner (1994) first showed that it is possible to assess implicit comprehension of false beliefs in 3-years-olds monitoring the direction of their gaze. Other researchers, using the violation of expectation paradigm have proven that also 15-month-old-children may attribute to an agent a false belief about the location of an object (Baillargeon et al., 2010).

These new results have generated what Perner and Roessler (2012) call a puzzle about belief. Why do 3-year-old children fail the classical false belief tasks whereas much younger children show the correct expectation in the spontaneous response tasks?

The question I intend to discuss here concerns the very existence of a puzzle. Are implicit and explicit false belief tasks comparable? To discuss this point let us analyze first the relationship between the false belief task and ToM.

## The Development of ToM and the False Belief Task

The importance attributed to the false belief task with respect to the development of ToM has been criticized in the past. For instance, Bloom and German (2000) argued that passing the false belief task requires other abilities besides ToM and conversely that ToM cannot be reduced to the ability to pass the false belief task. In a similar vein Apperly (2012) argues that there is more to ToM than having a conceptual grasping of mental states. In his view ToM is also a set of cognitive processes and a social competence attesting to individual variability; then no single task can be considered as the right “measure of ToM” across development.

In contrast, there are authors who still consider that the false belief task is a good indicator of explicit belief understanding. The hypothesis is that false belief comprehension is a step in a ToM scale including what children may know about persons and minds (Wellman and Liu, 2004). Cultural variation would result in differences in the sequence (Shahaeian et al., 2011). Variations in the sequence and acquisition times would interestingly characterize atypical populations like children with deafness, autism and Asperger syndrome (Peterson et al., 2012).

At the core of the present debate one point is central. What are we measuring with the different forms of false belief task that are in use? As we have seen the fundamental distinction regards implicit vs. explicit understanding. Anticipatory looking in young children allows inferring infants’ comprehension from their spontaneous behavior, while in the classical verbal tests children are asked to give an explicit answer about the false belief (for a review of the literature, see Low and Perner, 2012). It has been suggested that the implicit capacity shown by young children could have its basis in the human attitude to automatically encode others’ beliefs that would be active throughout development. This attitude has been shown both in 7-month-old infants and adults by Kovács et al. (2010) who argued that it could be part of a human-specific “social sense.” This work nicely fits in with the standpoint that the implicit and explicit false belief tests tap two different cognitive mechanisms and that perspective tracking is a process that it is often disrupted in various ways in the verbal versions of the task (Rubio-Fernández and Geurts, 2013). Interestingly, it has been shown that also in adults perspective tracking is a continuous process that can be disrupted by false belief questions (Rubio-Fernández, 2013). Thus, there is evidence in favor of the position that early understanding of belief is implicit while the classical false belief task requires explicit reasoning about actors’ reasons for how to act. It is the latter task that is not acquired before 4 years of age (Perner and Roessler, 2012).

The existence of two distinct systems was postulated by Apperly and Butterfill (2009). One system, efficient but limited and inflexible, would explain the ability shown by infants to deal with ToM tasks as well as social abilities of some non-human animals. The system constituted of mental concepts (desires and beliefs), would gradually develop in children allowing reasoning about others’ minds in a flexible but less efficient way. Adults would be equipped with both systems. This point of view is supported by evidence showing a task specific developmental continuity in false belief reasoning (Thoermer et al., 2012). An important role in the transcription of the first system into reasoning would be played by the emergence of language and executive functions.

Meta-analysis has shown no significant difference for false belief tasks types in their relation to language (Milligan et al., 2007). However, Helming et al. (2014) maintain that the analysis of the pragmatic framework of the test may elucidate the puzzle of belief ascription. Their thesis is that children’s second person engagement with the experimenter’s communicative action disrupts their ability to keep track of the content of the instrumental agent’s false belief. The cooperative perspective would prevail in the verbal task explaining children’s failure. Children would transform the experimenter test question into the question “Where *should* Sally look for her toy?”

Through the analysis of the literature it emerges that while at the beginning we had a definition of ToM and the false belief task was intended to ascertain its development now we are questioning the very definition of ToM. Thus the concept of implicit belief becomes central. What is an implicit belief and what is its relationship with ToM? How to avoid the risk of begging the question: ToM is what is measured by ToM tests? There is no demonstration that non-verbal and verbal false belief tests measure the same capacities, and that what we call implicit belief is comparable with explicit belief (San Juan and Astington, 2012).

## Intersubjectivity and ToM

My argument is that showing that infants have implicit grasp on others’ minds amounts to re-discovering intersubjectivity. Almost 40 years of studies on the development of intersubjectivity have shown that infants deal and communicate with other humans since birth (Trevarthen, 1998). It would be impossible to explain infants’ ability to deal with others if they had no grasp at all of what happens in their minds. Yet, this has little to do with what traditionally has been defined as ToM. My claim is that conflating intersubjectivity and ToM suggests a non-existing puzzle. The problem is the centrality attributed to the false belief task on the one hand and on the other hand the purported equivalence posed between the two forms of it, verbal and non-verbal. What Kovács et al. (2010) call a human-specific “social sense” has been shown for years in developmental research under the name of intersubjectivity. The development of the explicit capacity of reasoning about others’ mental states, i.e., ToM, is a “specialization” of human “social sense” that since the pre-school years humans *may* use to deal with particularly difficult situations.

One fundamental point of interest for developmental studies is the gap between how brilliant young children appear in some interactive situations and how ignorant of fundamental facts regarding other minds they turn out to be when tested in experimental situations.

Let us consider two cases of controversial interpretation of young children’s behavior, namely humor and deceit. The literature on intersubjectivity has shown that children before 2 years of age are able to participate in humorous interactions with adults and to engage in some forms of intentional falsifications of reality (Reddy, 2008). On the contrary in experimental situations these two forms of behavior are not shown to occur before 4/5 years of age. How may we explain this discrepancy? A possible answer is that in studies on intersubjectivity children are observed in the course of interactions and they show their intentionality and proficiency in engaging with others. Instead, in experimental situations children are requested either to explicitly manipulate others’ mental states or to have a judgment on the situation, i.e., to show what traditionally are considered as ToM abilities.

I take the case of humor. Young children in interactions with adults share situations of amusement. This means that there is a form of understanding that some acts are not serious: putting a breadbasket on one’s head is not the same as putting a spoon in one’s mouth. The first gesture makes others laugh while the second does not. Does this mean that young children “really know” what non-serious communication is? In order to investigate this we carry out experiments. For instance, a number of experiments have been made on irony comprehension. What do these experiments test? In general they test if children are able to comprehend that something has been said in a non-serious way, i.e., the real meaning of an ironic utterance but also if they understand the kind of act that has been produced, i.e., what being ironic means. Young children do not succeed in these tasks before 5/6 years of age. Thus the children that we observe in interaction are able to distinguish serious from non-serious situations in a rather appropriate way from a very young age, while in experiments children show that they do not know what being non-serious means till school age. Actually these experimental tasks are ToM tasks in the traditional definition and thus children have the traditional ToM results. In a study we designed an experimental task in which children had only to prove their comprehension of the communicative intention of ironic utterances, i.e., their non-literal meaning (Angeleri and Airenti, 2014). For instance, if a character said to another character who had just broken a plate: “Your mommy will be happy!” children were expected to understand that the intended meaning was that the mother would be upset. The goal was to have a comprehension task not burdened with ToM difficulties. In this condition we were able to show that even children as young as 3 years of age might understand the non-seriousness of an ironic utterance. In a sense we produced a kind of intermediate situation between using a communicative device in everyday communication and being able to explain what happens in another person’s mind in an experimental situation.

Thus, I argue that there is no puzzle. The so called explicit ToM is one of the aspects that intersubjective abilities may take in children not before 4 years of age and that evolves until adulthood. Younger children not only—as it is obvious—do deal with others but they do so in an effective manner *without* ToM.

We should come back to the fact that the false belief task has been devised in order to ascertain the development of the capacity of explicitly representing others’ beliefs. Designing false beliefs tasks that children may pass relying on those capacities that they normally use in their everyday behavior reverses the problem. But what does it prove with respect to ToM? 15-month-old children may pass the non-verbal false belief task but they are nevertheless unable to carry out or understand a deceit, to a find a good way to overcome a communicative failure, etc., i.e., to plan communicative acts that require representations of others’ beliefs. In sum, the problem of the development of ToM remains unaltered with the connected question regarding the role of language acquisition.

Humans are equipped from birth for interaction with others. This implies monitoring and adapting to others’ actions and participating in communicative exchanges. This is the *clever* part of young children’s behavior. However, some situations demand a more strategic thinking, i.e., reacting only after having reflected upon others’ mental states. For some basic interactive behaviors we can imagine two possible versions, one not implying ToM and one implying ToM. This applies for instance to failure. An infant happily communicates with her mother but if the mother just stops the interaction as it has been experimentally provoked in the *still face* situation or—with older children—interrupting a playing sequence, the child’s only possibility is protesting, manifesting discontent, diverting attention (Weinberg et al., 2008). Such a situation of unexpected behavior of the interlocutor can be handled by using ToM: Why does mom not smile at me anymore? or Why does mom not want to play with me anymore? The same applies to deceit. Young children may lie but many studies have shown that they do not plan real deceits and do not consider that others may be liars. Deceiving and discover possible liars demands ToM, i.e., reflecting on others’ beliefs (Airenti and Angeleri, 2011; Lee, 2013).

## Conclusion

In conclusion, the study of ToM was intended to understand how the capacity to represent mental states develops in children. The false belief task was designed to determine when and how this competence appears. Recently, the test itself has become the focus of the investigation. In this way we overlook the fact that the aim of ToM studies was to discover how children become able to perform acts as complicated as deceiving, discovering that others may be liars, or that in some situations lying is considered preferable than expressing real feelings. Actually, we have discovered something that we already knew, i.e., that even very young children have reasonable expectations with respect to others’ actions. This finding is consistent with the fact that in natural situations young children are able to deal with others rather successfully but does not improve our knowledge of ToM. I consider that using ToM terminology in this context is misleading. ToM is a particularly refined form of intersubjectivity and it is not intersubjectivity that has to be seen as a form of minimal ToM (Butterfill and Apperly, 2013). The question remains of explaining the mix of cleverness and candor so typical of young children, i.e., how ToM abilities transform early “social sense.”

### Conflict of Interest Statement

The author declares that the research was conducted in the absence of any commercial or financial relationships that could be construed as a potential conflict of interest.
